# Stardust and feminism: A creatureliness agenda

**DOI:** 10.3389/fpsyg.2022.1039210

**Published:** 2022-10-27

**Authors:** Nancy K. Dess

**Affiliations:** Department of Psychology, Occidental College, Los Angeles, CA, United States

**Keywords:** embodiment, gender, hegemonic human exceptionalism, creatureliness, new materialisms

## Abstract

People are living, breathing creatures. Dominant feminist discourses are situated within *hegemonic human exceptionalism* (HHE) which, by framing the body in terms of human forms of meaning-making and social life, eschews first-order embodiment (or *creatureliness*) as worthy of inquiry. Here, well-known reasons for avoidance of “the biological” are briefly summarized and an argument is advanced for meta-theoretical centering of creatureliness. A three-pronged agenda is proposed that embraces the creaturely body without the “-isms” (e.g., essentialism) and “-izings” (e.g., so-called “naturalizing”) that subvert feminist commitments. By unsettling HHE, executing the agenda would promote broader feminist coalitions and new scholarly collaborations aimed at fleshing out gender.

## Introduction

The universe began with a bang about 14 billion years ago. Transformations of stardust, energy, and gravity over the next 10 billion years yielded, among other things, Earth. After a billion years of cooling and declining acidity, life debuted, with every living thing from then on carrying hydrogen as old as the cosmos (Schrijver and Schrijver, 2020). Since then, a dazzling array of life forms has emerged and glided, trod, swum, sprouted, burrowed, and otherwise animated the globe.

Certain individuals spend time as a cell adrift in an aqueous milieu before metamorphosing into a bilaterally symmetrical tetrapod tethered to a host and then embarking on terrestrial life. There, they breathe air, eat, and defecate. They participate in a complex society in flexible ways contingent on, and constitutive of, body form and size, behavioral repertoires, social structure and composition, other life forms, and features of geography and climate (de Waal and Tyack, 2005).

These life forms—bonobos, elephants, humans, wolves, and others—live in ways that are enabled and constrained by their bodies, from the trillions of microbes each body hosts to overall mass, organ systems and specialized attunements to the world within and beyond the skin. They share some ways of living with blue whales, bluebirds, and bluebells. At the same time, each is in some ways distinctive at species, population, group, and individual levels and mutable on time scales from evolutionary to momentary. Sooner or later, they die.

What are the implications for gender studies of regarding humans not as “exceptional,” as minds housed in bodies that are inert input/output devices, as nodes or relations in networks, as wrought through human meaning-making, but rather as fully embodied creatures—as animals, as vertebrates, as mammals, as primates, as a particular sort of hominid? Taking up this question can spur scholarly and curricular innovation in service to feminism.

The rub: As Frost (2011) put it, “For feminist philosophers and theorists, the body as a living organism is a vexed object, so vexed, in fact, that in philosophical and theoretical work, it is often sidelined, bracketed, or ignored” (p. 69). Resolving that vexation can facilitate engaging the creaturely body without unduly constraining scholars’ prerogatives to focus on species and topics of interest.

## Rejecting the body

Unger (1979) argued that *gender* should be distinguished from *sex* because “‘sex’ implies biological mechanisms” (p. 1085). Unger did not refute human corporeality or biological sciences. The concern was with connotations of “the biological”: genetic determinism, reductionism, sex-typed behaviors as “hard wired.” The specter of an immutable sex binary was antithetical to feminist knowledge and commitments. Defining *gender* as sociocultural aimed to disrupt “-izing”– the binarizing, essentializing, and so-called “naturalizing” that *sex* evoked.

Unger’s call fostered vital change but reinforced dichotomies that undermine its purpose. Its “fatal flaw” was that “[I]n separating the biological from the social, it inadvertently reified both. If sex is to the biological as gender is to the cultural, the nature/nurture dichotomy is reinscribed” (Crawford and Fox, 2007, p. 483). The gender/sex, social/biological, nurture/nature dichotomies map onto each other and conjure others: fluid/immutable, mind/body, human/animal (e.g., Brescoll and LaFrance, 2004; Logan and Johnston, 2007; Overton, 2013). *Nurture/fluid* is to *mind/human* as *nature/immutable* is to *body/animal.* Thus, a gender [social/mind/human] cluster that enacts hegemonic human exceptionalism (HHE) is split from a sex [biological/body/animal] cluster. This G/S split reflects and perpetuates gender-and race-based segregation and stratification in academia as in society at large (Dess, 2022). Frost (2011) observed:

[F] eminists have been more comfortable with denaturalizing nature than with what we might call ‘deculturalizing culture’ – or admitting that matter or biology might have a form of agency or force that shapes, enhances, conditions, or delimits the agency of culture. Yet, this wary reluctance, understandable as it is given historical precedent, is structured by an understanding of causation that binds feminists to the binaries they have otherwise been deconstructing… the concern about unwitting essentialism is bound by the terms of Cartesian dualism that put rationality, freedom, and agency on one side of an ontological divide and matter, passivity, and determinism on the other. (p. 76)

To realize feminist goals, the G/S split and its proxies must be replaced with richer formulations.

## Reclaiming the body

The time is ripe to leverage the work of feminist biologists (Anne Fausto-Sterling, Patricia Adair Gowaty, Sarah Blaffer Hrdy, and others) and *new materialists* in psychology and other disciplines (e.g., Grosz, 1994; Alaimo and Hekman, 2008; Frost, 2011) into a paradigm shift away from HHE. Here, three proposals are offered in service of that goal.

### Adopt language reforms

The term *biological* should be used to refer to academic enterprises. This shift attends to the suffix of bio*logy*, the *study* of living things, and what bio*logists* do. This institutional meaning is routinely conflated with the stuff of life at low levels of organization (e.g., cells, biochemicals). Biologists also take up how life is organized at higher levels (e.g., sociality, ecology), but such work is less schematic for “the biological.” The topics that draw the biologist’s eye and how they are studied vary wildly. Dumping them into a bucket labeled “the biological” and distinguishing them from “the social” makes no sense, especially given that the latter lacks institutional meaning comparable to *biological*. More precise terms, such as *genes* or *nervous system*, should be used when referring to topics in biological sciences. Similarly, sociocultural constructs should be identified with specificity (see Magnusson and Marecek, 2018, on unpacking “the social”), not termed *nonbiological*. Nothing about life is *nonbiological* in the sense that cells are not involved, and the institutional meaning—to wit, “things biologists do not study”—is hopelessly vague.

Replacing “the biological vs. the social” with a bigger, better lexicon will make the G/S split obsolete. Because this reform explodes exceptionalist dichotomies that remove humans from nature (natural/artificial, nature/people, “humans and animals”; Dess and Chapman, 1998), it will neutralize “naturalizing.” How to repurpose the terms *gender* and *sex* (again) will need to be negotiated.

### Adopt an updated view of biological sciences

Transformations in biology—including epigenetics, developmental psychobiology, neuroscience, and evolutionary theory—debunk the linkage of “the biological” to immutability and system justification. The working vocabulary includes terms such as *contingent*, *probabilistic*, *dynamical*, and *path-dependent*. Genes are not a “blueprint”: They are expressed variably and contingently on the environment, sometimes transgenerationally (Ghai and Kader, 2022). Similarly, developmental trajectories are viewed as sensitive to *in utero* and postnatal contexts. No vertebrate brain is “female” or “male”; brains develop and function flexibly, are largely the same within a species, and are conceptualized as a mosaic (Joel, 2021), inherently bisexual (Crews, 2012), and experience dependent (Hines, 2018).

Today’s evolutionary theory likewise is no tale of genetic determinism or hard wiring. Rejection of such simplistic notions is not new: Decades ago, seminal work on human mating strategies (Buss and Schmitt, 1993) refuted the notion that evolution yielded intractable sex differences, showing instead that “Mate preferences, far from being impervious to varying circumstances, are highly sensitive to temporal contextual conditions” (p. 230). The Integrated Synthesis (Pigliucci and Müller, 2010), which incorporates culture, multi-level selection, plasticity, and environmental control of gene expression, increasingly undergirds evolutionary theory in psychology. This project is actively underway (Narvaez et al., 2022) and would benefit from more feminist voices.

The call to overcome “biophobia” has come from psychologists (Salk and Hyde, 2012), sociologists (Freese et al., 2003), political scientists (Frost, 2011), and biologists [cf. Fausto-Sterling’s observation that “Culture shapes bones,” Fausto-Sterling, 2005, p. 1491]. Movement toward that end in gender scholarship and teaching must accelerate for the body to become more than inert matter or human meaning.

### Adopt inclusive meta-theoretical frameworks

Grappling with massively complex phenomena requires a large tool kit and some division of labor. How can labor over *gender* be divided without devolving to proverbial blind men fighting over what an elephant is? A modest step would be adoption of meta-theoretical frameworks that locate scholarly enterprises in relation to each other. Two such frameworks distinguish *levels of organization* from macro to micro and *time scales* from deep time to nanoseconds (e.g., Bronfenbrenner, 1992; Li, 2003; Overton, 2013; Dess, 2022). Although the number and labeling of levels/scales are somewhat arbitrary, getting beyond two levels/scales prevents binaries, and labels can be negotiated. The point is to foster a shared understanding that although life exists at all levels of organization and on all time scales, a scholarly lens necessarily focuses on certain levels and scales. Such an understanding transforms “turfs” and “siloes” to equal-status locations in relation to each other, with some common and some distinctive interests and commitments.

A crucial premise is that everything about bodies at every level of organization is mutable on some time scale(s). Far from an exception, gender/sex expressions at all levels vary across time and contexts in many species (cf. Roughgarden, 2004). Anybody who regards gender/sex comparatively will *anticipate* the mixy/matchy, blendy/bendy nature of gender/sex in *Homo sapiens*, a globe-roaming, omnivorous, time-traveling, symbol-using, obligately social primate (de Waal, 2022).

The wide world of gender/sex features both *variation* and *constraint*. An example at the subcellular level: allosomes (traditionally called *sex chromosomes*). An XX/XY scheme is not necessary for reproduction, and being homogametic (e.g., XX) is not “essentially female.” Across deep time, allosomes have been a flexible tool in the adaptation kit. Vertebrate species have 0–10 allosomes. In some reptiles and birds, the heterogametic individuals lay eggs, and some can reproduce either sexually or parthenogenetically. Moreover, allosomes do not determine sex-typed mating behavior. Every *A. uniparens* lizard, for instance, has XXX allosomes, is a parthenogen, and displays mating behaviors typical of egg-and sperm-producing members of related species (O’Connell and Crews, 2022).

On an evolutionary timescale, then, allosomes are flexibly related to bodies and behavior. On a developmental timescale, gene expression is probabilistic and context dependent so, for example, an XY human zygote will not necessarily grow up to have a penis, produce sperm, identify as a *man* or *rajul*, or in myriad other ways be like other XY individuals. However, those forms of contingency and mutability are different from how allosomes vary within a species on shorter time scales. Regardless of identity or intrauterine or cultural environment, at some stage every somatic cell in a human born alive has 1–5 allosomes, with XX and XY being the highest-frequency variants (~99%). An XY zygote will not grow a uterus—regardless of sex assigned at birth and adult identity—and will not gestate a fetus, seek an abortion, or die giving birth. Human allosomes do not generate two human “kinds,” nor do they determine identity, behavior, lived experience, or social organization, validate treating gender as binary, or justify injustices. But they are matter that matters.

Using levels/scales frameworks to grapple with *gender* is compatible with conceptualizing bodies in symbolic, discursive, and cultural terms. In fact, it compels doing so. But it exposes the insufficiency of those (and any other) conceptualizations by illuminating domains of inquiry that are not tractable from those standpoints. These frameworks also illuminate paths to integration. Events at various levels of organization are presumed to interact through recursive loops on various time scales, and those loops pull for “cross-country” work. Research by Lisa Diamond, Sari van Anders, and Felicia Pratto exemplifies effective use of multi-level/scale, dynamical systems, and multi-method approaches to the complexity of gender.

## Discussion

Reclaiming the body will allow feminists to take up in a more fulsome way the role of unmediated corporeal realities in individual and social well-being. For instance, it affords seeing the project of overturning *Roe v. Wade* as partly about *women* and partly about *members of a mammalian species able to gestate a fetus*. Reproductive rights (or lack thereof) have everything to do with the traditional stocks-in-trade of feminists, such as challenging patriarchy, championing sexual and economic autonomy, and interrogating how *family* is defined. They also have everything to do with risks borne only by individuals on the receiving end of internal fertilization and gestation. If, like salmon and sparrows, humans laid eggs, gendering would play out differently in policy, practice, and everyday life.

If too much attention to reproductive biology smells of essentialism, too little attention seems awfully bourgeois given class, national, and racial disparities in maternal and infant mortality. Furthermore, a compelling case can be made that patriarchal control of gametic female reproductive prerogatives has dire fitness consequences for *all* stakeholders (Gowaty, 2020). Theory and policy arguments incorporating first-order embodiment can usefully complement those rooted in discursive practices or disembodied rights.

To be clear, defensively burying the creaturely body is not unique to mainstream feminism. Doing so pervades academia (Dess, 2022), perhaps due to a core implication of corporeality: mortality (Solomon, 2020). A silver lining of HHE’s pervasiveness, however, is that rejecting it has tremendous potential to positively impact academic culture and society at large. As David Abram observes in *Becoming Animal* (Abram, 2010):

Becoming earth. Becoming animal. Becoming, in this manner, fully human… Corporeal life is indeed difficult… Thus do we shelter ourselves from the harrowing vulnerability of bodied existence. But by the same gesture we also insulate ourselves from the deepest wellsprings of joy… Awakening to citizenship in this broader commonwealth… has real ramifications for how we humans get along with one another. It carries substantial consequences for the way a genuine democracy shapes itself – for the way that our body politic *breathes* (emphasis original; pp. 3-9).

The reforms proposed here transform the term *gender* into an inclusive rubric that subsumes *sex* – to wit, a large repertoire of loosely associated characteristics that vary and are constrained at various levels of organization and on various time scales. Comprehensive understanding may elude, but a shared vision of an expanded scope of inquiry that is not rooted in HHE can move feminist scholarship in new directions.

## Author contributions

The author confirms being the sole contributor of this work and has approved it for publication.

## Conflict of interest

The author declares that the research was conducted in the absence of any commercial or financial relationships that could be construed as a potential conflict of interest.

## Correction note

This article has been corrected with minor changes. These changes do not impact the scientific content of the article.

## Publisher’s note

All claims expressed in this article are solely those of the author and do not necessarily represent those of the affiliated organizations, or those of the publisher, the editors and the reviewer. Any product that may be evaluated in this article, or claim that may be made by its manufacturer, is not guaranteed or endorsed by the publisher.
